# G-Protein-Coupled Receptor Gpr17 Regulates Oligodendrocyte Differentiation in Response to Lysolecithin-Induced Demyelination

**DOI:** 10.1038/s41598-018-22452-0

**Published:** 2018-03-14

**Authors:** Changqing Lu, Lihua Dong, Hui Zhou, Qianmei Li, Guojiao Huang, Shu jun Bai, Linchuan Liao

**Affiliations:** 1 Department of Forensic Analytical Toxicology, West China School of Basic Medical and Forensic Medicine, Sichuan University, Chengdu, 610041 Sichuan China; 2 Department of Anatomy, West China School of Basic Medical and Forensic Medicine, Sichuan University, Chengdu, 610041 Sichuan China; 3 Department of Pediatrics, West China Second University Hospital, Sichuan University, Chengdu, Sichuan Province China

## Abstract

Oligodendrocytes are the myelin-producing cells of the central nervous system (CNS). A variety of brain disorders from “classical” demyelinating diseases, such as multiple sclerosis, stroke, schizophrenia, depression, Down syndrome and autism, are shown myelination defects. Oligodendrocyte myelination is regulated by a complex interplay of intrinsic, epigenetic and extrinsic factors. *Gpr17 (*G protein-coupled receptor 17) is a G protein-coupled receptor, and has been identified to be a regulator for oligodendrocyte development. Here, we demonstrate that the absence of *Gpr17* enhances remyelination *in vivo* with a toxin-induced model whereby focal demyelinated lesions are generated in spinal cord white matter of adult mice by localized injection of LPC(L-a-lysophosphatidylcholine). The increased expression of the activated form of *Erk1/2* (phospho-*Erk1/2)* in lesion areas suggested the potential role of *Erk1/2* activity on the *Gpr17*-dependent modulation of myelination. The absence of *Gpr17* enhances remyelination is correlate with the activated *Erk1/2* (phospho-*Erk1/2*).Being a membrane receptor, *Gpr17* represents an ideal druggable target to be exploited for innovative regenerative approaches to acute and chronic CNS diseases.

## Introduction

Oligodendrocytes are the myelin-producing cells of the central nervous system (CNS), and as such, wrap layers of lipid-dense insulating myelin around axons^1^. Mature oligodendrocytes have also been shown to provide metabolic support to axons through transport systems within myelin, which may help prevent neurodegeneration^2^.

Oligodendrocytes are generated from oligodendrocyte precursor cells (OPCs), which migrate to and colonize the brain’s white matter (and sometimes gray matter as well) and spinal cord^3–5^. This process is tightly controlled not only by a complex intrinsic oligodendrocyte differentiation program^6^, but also by external reciprocal signaling processes such as the degree of neuronal differentiation^7^. Previous studies have demonstrated that adult SVZ progenitors can generate new OPCs/oligodendrocytes after demyelinating lesions of the corpus callosum^8,9^, seizures^10^, or stroke^11,12^. These OPCs may participate in myelin repair after injury^13,14^.

OPCs respond to demyelinating injury by first undergoing activation, colonization of the demyelinated area by proliferation and migration, and eventually differentiation into new myelin-forming oligodendrocytes^15,16^. Critical to this process is the switch from a proliferative/migratory state to the exiting from the cell cycle and differentiation into a nondividing, nonmigratory mature oligodendrocyte.

Identifying pathways and transcription factors involved in the regulation of OPC differentiation in myelination and especially remyelination that can potentially be manipulated pharmacologically represents a critical task in the development of new therapies for enhancing endogenous remyelination and thus axonal protection in MS and other myelin disorders^17^. At present, the factors that promote the initiation of OPC differentiation and overcome the block for successful remyelination in demyelinating diseases are poorly defined^18^.

*Gpr17* is an orphan G-protein-coupled receptor that responds to both uracil nucleotides and cysteinyl leukotrienes (cysLTs)^19,20^. Endogenous ligands of *Gpr17*, such as UDP glucose and cysLTs, have been identified, and synthetic ligands, such as MDL29951 and pranlukast, have been developed to activate or antagonize *Gpr17* activity, respectively^19,21,22^.

Activation of *Gpr17* signaling upregulates the expression of a differentiation inhibitor, *ID2*, and promotes the nuclear translocation of *ID2* and *ID4*^23^. Overexpression of *Gpr17* in the oligodendrocyte lineage causes defects in myelinogenesis in transgenic mice, and *Gpr17* knock-out mice exhibit precocious myelination in the spinal cord at the neonatal stage^23^. The hypothesis that activation of *Gpr17* delays oligodendrocyte maturation is supported by recent findings that *Gpr17* desensitization by G-protein receptor kinase phosphorylation and subsequent internalization are necessary for terminal differentiation of OPCs^24^. Furthermore, *Gpr17* has been shown to negatively regulate oligodendrocyte differentiation via the inactivation of intracellular protein kinase A (PKA) and cAMP-activated GTP exchange factor Epac1^25^. In addition to the regulation of normal oligodendrocyte development, *Gpr17* also functions as a sensor for extracellular damage signals under pathological conditions such as ischemia and brain trauma^21,26–28^. Remyelination is more rapid in *Gpr17* knockout mice than in wild-type mice after a lysolecithin injection in the corpus callosum^29^. *Gpr17* antagonism results in structural and functional rejuvenation of aged brains, suggesting a promising clinical application for a *Gpr17*-based intervention^30^.

In the present study, we chose the L-a-lysophosphatidylcholine (LPC) lesion as a model to examine the temporal response and transcription factor expression of endogenous OPCs following demyelination. We elucidated the role of *Gpr17* in the survival and differentiation of oligodendrocytes in response to spinal cord demyelinating injury. The absence of *Gpr17* enhances remyelination is correlate with the activated Erk1/2 (phospho-Erk1/2).

## Results

### *Gpr17* expression gradually increases during LPC induced demyelination

**Figure 1:**
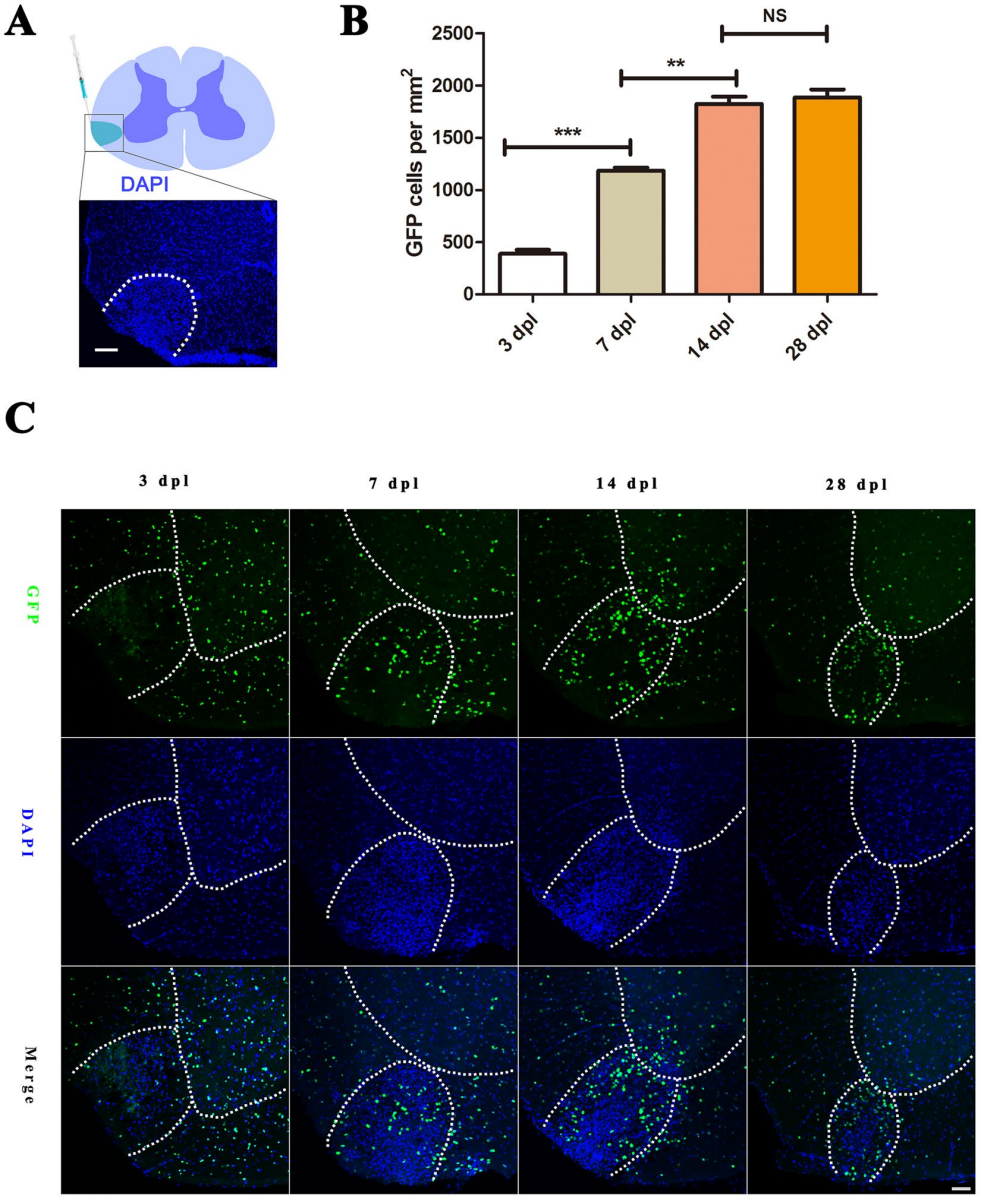
*Gpr17* expression gradually increases during LPC induced demyelination. (**A**) The location of LPC-induced lesion (DAPI counterstaining, dashed lines in white matter) in the spinal cord. (**B**) Representative expression of *Gpr17*, visualized by the expression of the reporter protein GFP, in the LPC-induced demyelinating lesions (demarcated with dashed lines in white matter) in spinal cords of 8-week-old *Gpr17*^–/–^mice at 3 dpl,7 dpl, 14 dpl and 28 dpl. (**C**) Quantification of the numbers of GFP at 3 dpl,7 dpl, 14 dpl and 28 dpl; We compared GFP of the Gpr17^−/−^ as follows,3 dpl vs. 7 dpl,7 dpl vs. 14 dpl, 14 dpl vs. 28 dpl. Image J was used to measure area of the lesion, count the cells. Sections were taken from the center of each lesion to control for lesion variability. White dashed line demonstrates lesion borders. Student’s t-test, Data are presented as Mean ± SEM, Error bars indicate SEM, **P < 0.01, ***P < 0.001; n = 3 animals for each genotype, Scale bar: (**A**) 25 μm; (**B**) 50 μm. LPC, L-a-lysophosphatidylcholine, Dpl: days post lesion; Ctrl, control; WM:white matter; GM:gray matter.

*Gpr17*^−/−^ mice was deleted the entire *Gpr17* coding region, replaced with histone 2b–fused GFP (h2b-GFP) to trace individual endogenous *Gpr17*-expressing cells^23^. To assess the function of *Gpr17* in remyelination *in vivo*, we employed the LPC-induced demyelination in white matter of spinal cord. Stereotaxic injection of LPC into the adult spinal cord results in selective and focal myelin loss with minimal axonal damage in adjacent cells and axons, induces subsequent remyelination within 4 weeks^31–33^. The relatively short duration of the experiments and the easy analysis of the demyelinated area make it a convenient model to study demyelination ⁄ remyelination processes^34^. Myelin regenerates through an OPC recruitment phase at 7 days post lesion (dpl) and an oligodendrocyte regeneration and remyelination phase at 14 dpl^35^. LPC was injected into the ventrolateral column of 8-week-old male wild type (control), *Gpr17*^+/−^(control)^23^, and *Gpr17*^−/−^ mice from the same litter to trigger demyelination(Fig. 1A). Increased cell density (as for instance shown by DAPI staining) represents the LPC lesions*.Gpr17* expression, indicated by the expression of the reporter GFP, is almost absent at 3 dpl, but gradually increases at 7 dpl, 14 dpl and reaches its peak at around14 dpl. At 7 dpl, *Gpr17* is observed in the perimeter of the lesion and outside the demyelination area, excluding the pia border. At 14 dpl, the expression of *Gpr17* appeared to be more densely distributed than in adjacent normal white matter, reaches its peak around14 dpl (Fig. 1B,C), which is consistent with previous studies demonstrating increased oligodendrocyte densities in remyelinating regions^36^.

### Loss of *Gpr17* promotes remyelination after LPC -induced demyelination in the CNS

**Figure 2:**
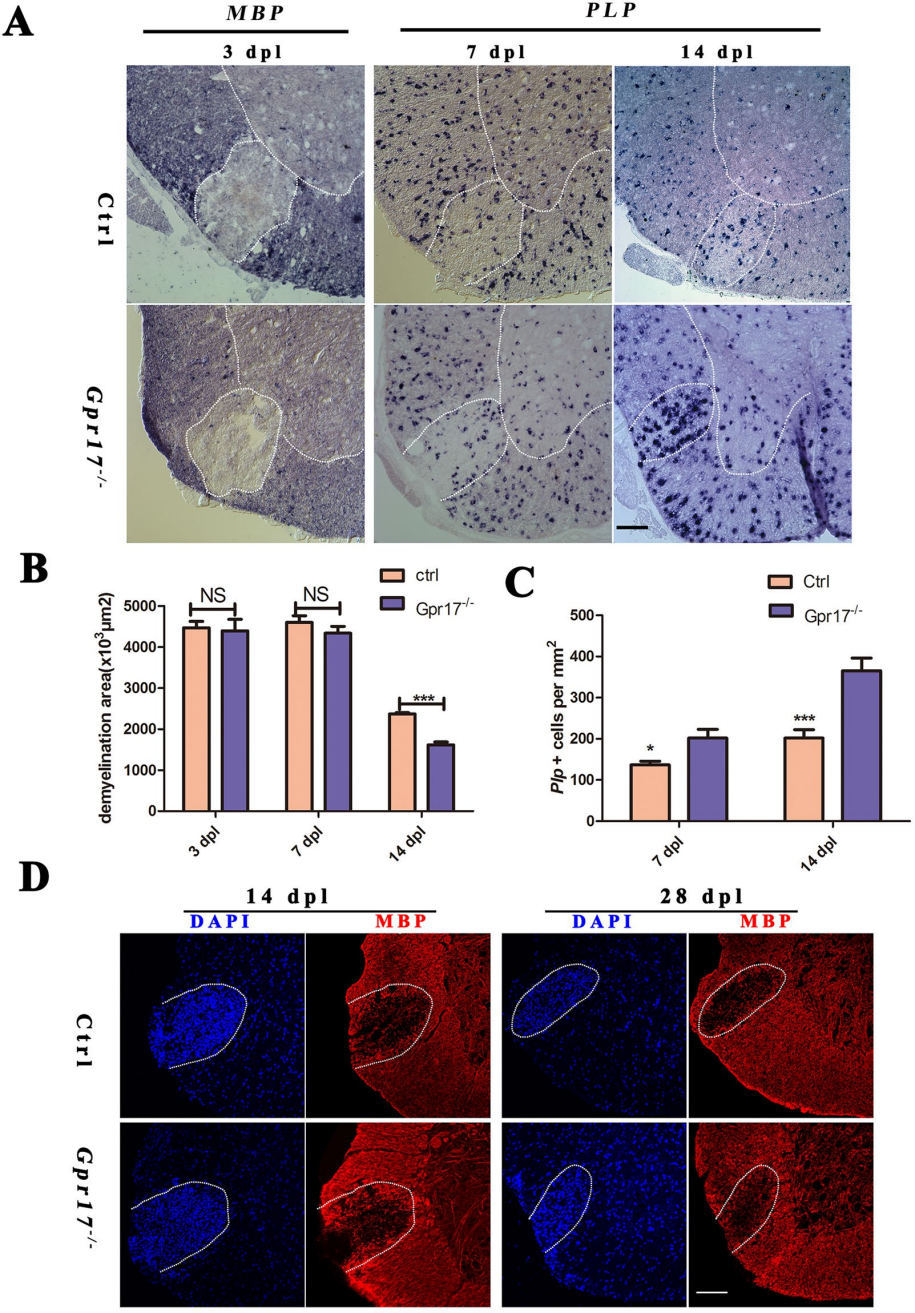
*Gpr17* ablation promotes remyelination in LPC-induced demyelinating animal model. (**A**) *In situ* hybridization analysis of *MBP* and *PLP* in the lesion regions (demarcated with dashed lines) at 3 dpl,7 dpl and 14 dpl in spinal cords of 8-week-old control and *Gpr17*^−/−^ mice; n = 3 animals for each genotype. (**B**) Quantification of the areas of lesion regions (demarcated with dashed lines) at 3 dpl,7 dpl and 14 dpl in spinal cords of 8-week-old control and *Gpr17*^−/−^ mice. (**C**) Quantification of the numbers of *PLP* in the lesion regions (demarcated with dashed lines) at7 dpl and at 14 dpl. (**D**) Immunostaining of *MBP* in lesion regions at 14 dpl and 28 dpl in spinal cords of 8-week-old control and *Gpr17*^−/−^ mice; n = 3 animals for each genotype. Data are presented as Mean ± SEM. *P < 0.05, ***P < 0.001; Student’s t-test. Scale bars, (**A**,**D**)100 μm.

**Figure 3:**
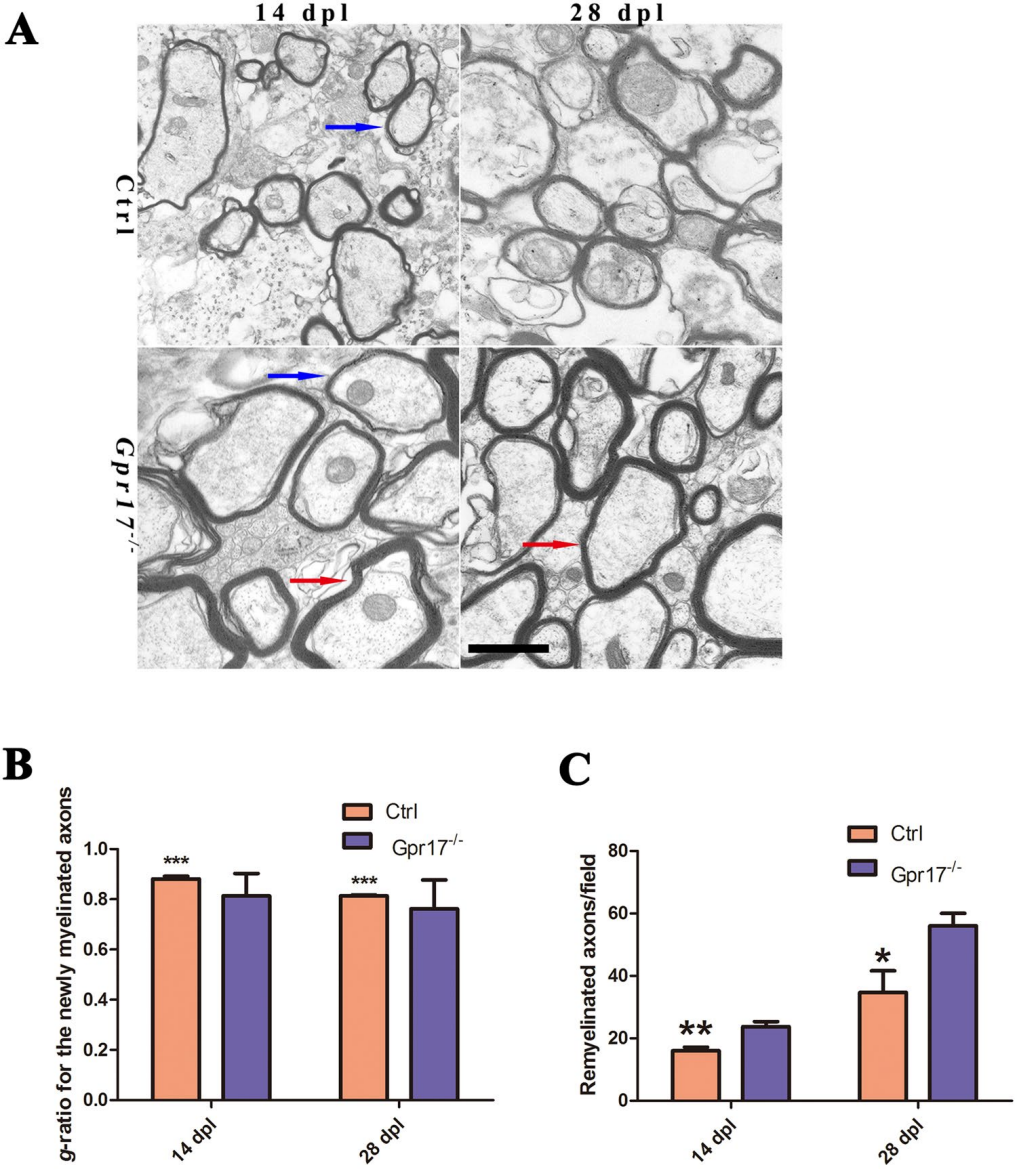
*Gpr17* ablation promotes remyelination in LPC-induced demyelinating animal model. (**A**) Representative electron micrographs of spinal cords of 8-week-old control and *Gpr17*^−/−^ mice at 14 dpl and 28 dpl, n = 3 animals for each genotype. Blue arrow indicates the newly formed thin myelin sheath. Red arrow indicates hypermyelination of axons. (**B**) Quantification of g-ratio of newly myelinated axons with diameter of 1 μm in spinal lesions of 8-week-old control and *Gpr17*^−/−^ mice at 14 dpl and 28 dpl; P < 0.001, Student’s t-test. (**C**) Results of morphometry to quantify remyelination at 14 dpl and 28 dpl (see *Materials and Methods*). Data are presented as Mean ± SEM. *P < 0.05, **P < 0.01, ***P < 0.001; Student’s t-test. Scale bar, (A) 1 μm.

Previously, the analysis of *Gpr17*^−/−^mice showed that *Gpr17* functioned as a cell-intrinsic factor that blocked oligodendrocyte terminal differentiation^23^. To determine whether the loss of *Gpr17* facilitates remyelination after injury and accelerates the recovery of injured myelin sheaths, we analyzed the expressions of two myelin genes, Myelin basic protein *(MBP*) and proteolipid protein (*PLP*), in the lesions at 3dpl, 7dpl and 14 dpl. *MBP*, a maturing oligodendrocyte marker, which labels both premyelinating and myelinating oligodendrocytes. *PLP* exhibits transcriptional upregulation during differentiation from the immature progenitor stage to the mature oligodendrocyte stage^37^. At 3 dpl, both Gpr17 null and control mice exhibited comparable lesions with very little MBP mRNA expression as detected by *in situ* hybridizations within the lesion, indicating a similar loss of preexisting myelin and no remyelination occurred at this stage (Fig. 2A*)*. However, at 7dpl and 14 dpl, the expression of *PLP* was comparable between *Gpr17* null mice and control littermates (Fig. 2A,C). At 14 dpl and 28 dpl, *Gpr17*^−/−^ mouse showed more profound expression of *MBP* in lesion region in comparison to control group (Fig. 2D). The average lesion size of *Gpr17*^−/−^ was significantly smaller compared with control group (Fig. 2A,B). Importantly, as indicated by electron micrographs, a great number of hypermyelination of axons were detected in the lesions of *Gpr17*^−/−^ mice than in controls (Fig. 3A). The thickness of newly generated myelin sheaths around axons was significantly increased in the gpr17 null mice (Fig. 3A). Myelinogenesis was comparable between control and *Gpr17*^−/−^ mice as indicated by the integrity of the myelin sheath ultrastructure in the lesion (Fig. 3B,C). There were more remyelinated axons in *Gpr17*^−/−^ mice both at 14 dpl and 28 dpl (Fig. 3C). These observations indicate that loss of *Gpr17* promotes remyelination after demyelinating injury. Despite the discrepancy in the onset of remyelination, both the mutant and the control mice do not exhibited complete remyelination at 28 dpl (Fig. 2D, Supplematary data S1).

### Enhanced oligodendrocyte differentiation was recruited toward demyelinated lesions

**Figure 4:**
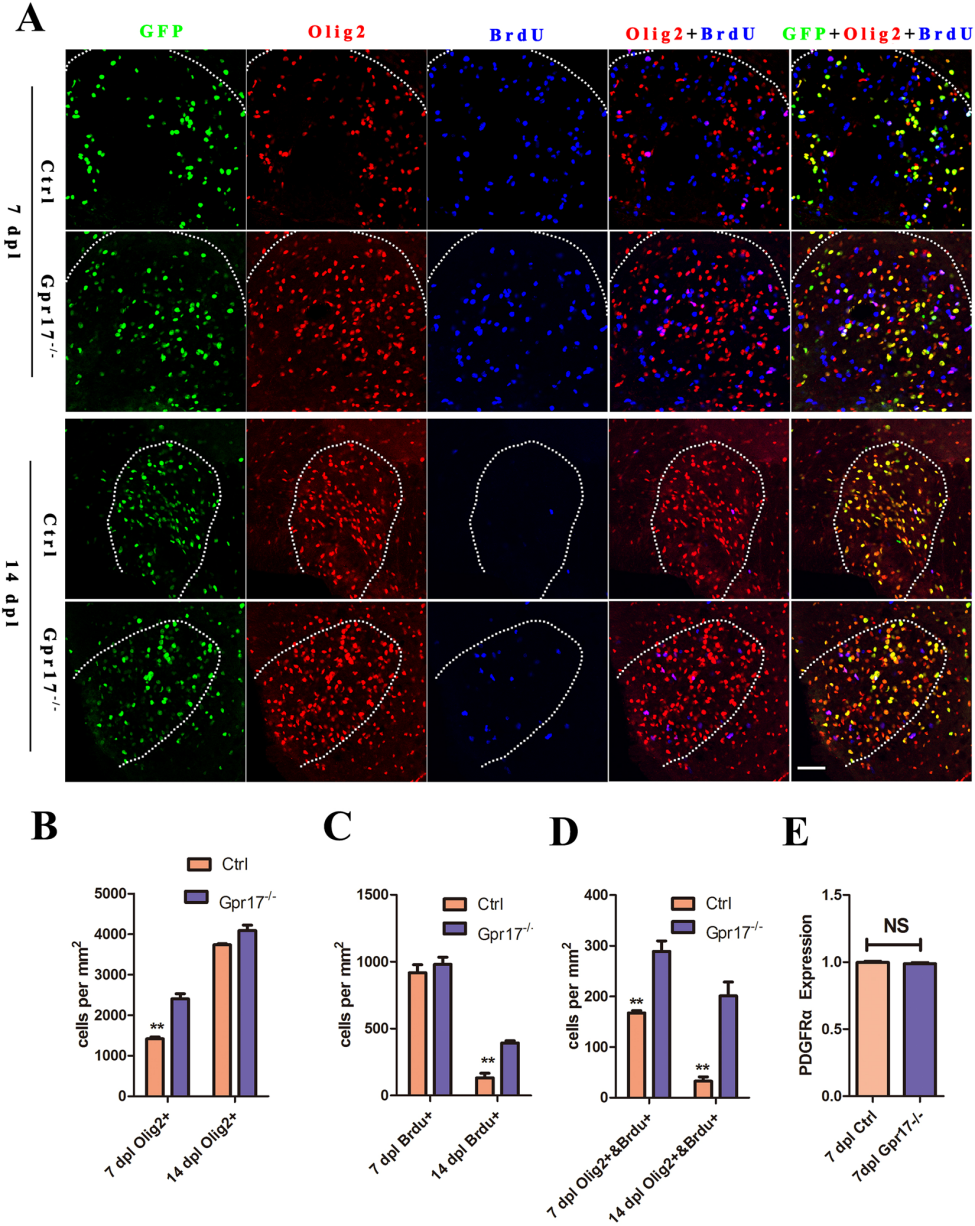
The expression of Olig2 proliferation was increased in lesion regions in *Gpr17*^*−/−*^ mice. (**A,B**) and (**C**) Immunostaining and quantification of for expression of OL lineage cells marker Olig2 and BrdU in lesion regions at 7 dpl and 14 dpl in spinal cords of 8-week-old control and *Gpr17*^*−/−*^ mice; n = 3 animals for each genotype. White dashed line demonstrates lesion borders. (**D**) Quantitative real-time PCR (qRT-PCR) analysis of OPCs marker PDGFRa expression in lesion regions. n = 3 animals for each genotype. Student’s t-test. Data are presented as Mean ± SEM. **P < 0.01; Scale bar: 50 μm.

During the remyelination process, OPCs are recruited toward demyelinated lesions^38^. *Olig2*, which marks a subset of glioblasts and maturing OPCs, is known to be critical for oligodendrocyte specification and differentiation^39,40^. We performed immunohistochemistry for *Olig2* at 7dpl and 14dpl. To investigate whether there were changes in the proliferation of *Olig2* + cells in response to injury in the mutant mice, we administered the thymidine analog BrdU (5-bromo-2′-deoxyuridine, Sigma-Aldrich,) to mice 2 hours before sacrifice, thereby labeling cycling cells across this time period via its incorporation into DNA during replication. We found that the number of BrdU+/Olig2 + cells in the lesion ventrolateral column increased at 7dpl and 14 dpl (Fig. 4A–D), indicating significant increase in *Olig2* + cell proliferation.

To investigate whether there were changes in OPCs in response to injury, we performed quantitative real-time PCR (qRT-PCR) for OPC marker *PDGF receptor α*. (*PDGFRα*). The expression was not significantly different between the two genotypes at 7dpl (Fig. 4E), suggesting that the generation of OPCs was not affected by the deletion of *Gpr17*.

**Figure 5:**
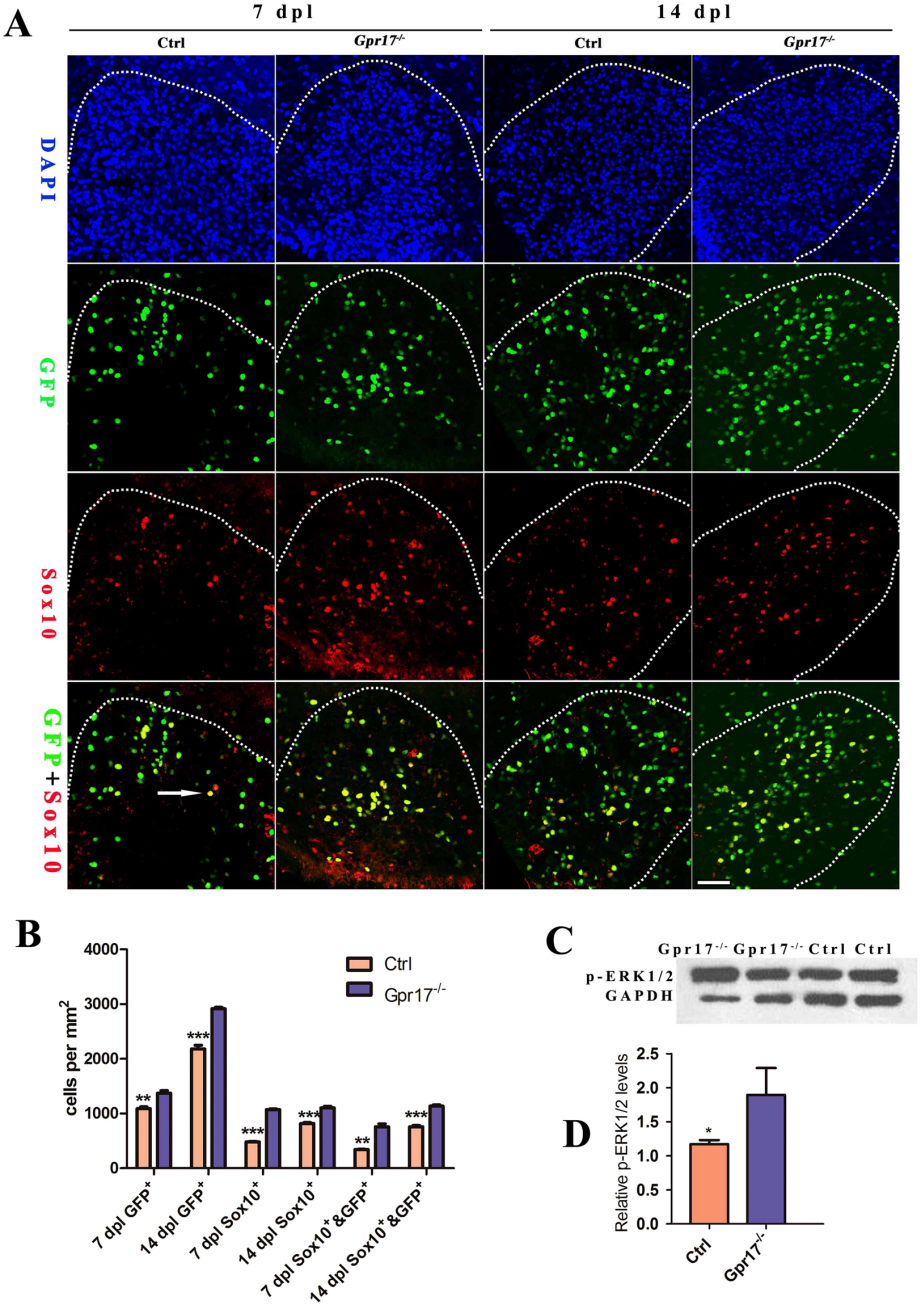
*Gpr17*-dependent inhibition of oligodendrocyte differentiation is mediated by Erk phosphorylation. (**A** and **B**) Immunostaining and quantification of expression of oligodendrocyte differentiation-promoting factor Sox10 and GFP in lesion regions at 7 dpl and 14 dpl in spinal cords of 8-week-old control and *Gpr17*^−/−^ mice; n = 3 animals for each genotype. Arrow indicates double labeled with sox10 and GFP. White dashed line demonstrates lesion borders. (**C** and **D**) Western blot analysis of p-Erk1/2 expression in lesion regions at 14 dpl in spinal cords of 8-week-old control and *Gpr17*^−/−^ mice; n = 4 animals for each genotype. Student’s t-test. Data are presented as Mean ± SEM. *P < 0.05, **P < 0.01, ***P < 0.001; Scale bar: 50 μm.

*Sox10* is an oligodendrocyte differentiation-promoting factor, required for terminal differentiation of the oligodendrocyte lineage^41–43^. In *Sox10*-deficient mice progenitors develop, but terminal differentiation is disrupted^43^. We performed immunohistochemistry for Sox10. In contrast, *Gpr17*^−/−^ mice showed a dramatic increase in *Sox10* + immunoreactivity in the demyelinated area, particularly around the edges of the lesion (Fig. 5A,B). Small amounts of *Sox10* + immunoreactivity were occasionally seen scattered throughout the lesion in control mice (Fig. 5A,B). Taken together, the enhanced oligodendrocyte differentiation was recruited toward demyelinated lesions, we hypothesized that the recruitment is correlates with *Sox10*.

### *Gpr17*-dependent inhibition of oligodendrocyte differentiation is mediated by *Erk* phosphorylation

The extracellular signal-regulated kinases, *Erk1* and *Erk2*, are prototypic members of the mitogen activated protein kinase (*MAPK*) family^44^. *Erk1/2* activation has been studied extensively in oligodendrocyte development, where it promotes differentiation and increased myelin thickness^45^. In mice, genetic loss of *Erk1/2* in the oligodendrocyte lineage results in normal numbers of OPCs and oligodendrocytes but widespread hypomyelination, while constitutive activation of *Erk1/2* results in a profound increase in the extent of remyelination after toxin-induced demyelinating injury^46,47^. To explore whether the effect of GPR17 on oligodendrocyte differentiation is influenced by *Erk1/2*, we performed Western Blotting of phospho-Erk1/2 at 14 dpl. We detected increased expression of phospho-Erk1/2 in *Gpr17*^−/−^ mice (Figs 5C,D, S3). Taken together, our results indicate that the inhibitory action of *Gpr17* in oligodendrocyte maturation (that is, late-stage differentiation) and myelination are correlated with *Erk1/2* pathway.

## Discussion

Rushton (1951) proposed that for a given axon diameter, there is a specific myelin thickness that maximizes conduction velocity^48^. Alterations in this ratio affect the speed of nerve impulse transmission and thus the timing of neuronal signals, potentially leading to aberrant circuit connectivity, and/or rendering axons vulnerable to damage^47^. A better understanding of the molecular mechanisms and signaling pathways that drive the process of myelin sheath formation is therefore important for the development of novel therapeutics designed to target remyelination^45^. Here, we identify *Gpr17* as a negative regulator of oligodendrocyte maturation (late-stage differentiation) in the adult mouse spinal cord after demyelinating injury.

To test if the absence of *Gpr17* enhances remyelination *in vivo*, we used a toxin-induced model whereby focal demyelinated lesions are generated in spinal cord’s white matter of adult mice by localized injection of LPC. In animal lesions, demyelination is completed within four days, after which OPCs are recruited into the lesion. Widespread remyelination does not normally commence until 14–21 days post lesion (dpl), which provides a defined window from days 4–14 to test the efficacy of drugs to enhance the extent and rate of remyelination^31^. *Gpr17* expression, which is almost absent at 3 dpl, gradually increases at 7 dpl and 14 dpl, reaches its peak around14 dpl, this result is consistent with previous studies indicated *Gpr17* almost absent in early OPCs, gradually increases in more mature precursors, reaches a plateau in immature/pre-oligodendrocytes, and then gradually decreases during terminal differentiation^23,27,49^. In various *in vivo* neurodegenerative models characterized by myelin loss (stroke, trauma, demyelination, and experimental autoimmune encephalomyelitis), *Gpr17* was abnormally up-regulated in OPCs around lesion sites^21,27,50^. In rat brain, *Gpr17* is expressed in OPCs and not in mature oligodendrocytes^49^. The deletion of *Gpr17* resulted in an earlier onset of remyelination in mice, which is confirmed by the expression of myelin gene expression *MBP* and *Plp* at 7dpl, 14 dpl, 28 dpl, and myelin sheath ultrastructure at 14 dpl and 28 dpl (Figs 2A,D, 3A). Which is consistence with previous research showing that remyelination is more rapid in *Gpr17* knockout mice than in wild-type mice in the corpus callosum^29^.

Myelination is mediated by oligodendrocyte precursor cells (OPCs) that are widely distributed throughout both the gray and white matters of the CNS throughout life^51,52^. Adult-born oligodendrocyte can continue to proliferate and produce compact myelin^51^. Although a significant fraction of OPCs remains undifferentiated, particularly in gray matter, many will eventually differentiate to become myelinating oligodendrocytes^53^.Development of the oligodendrocytes lineage is controlled by an intricate regulatory transcriptional network that includes multiple inhibitory and stimulatory factors^54,55^. We found that the absence of *Gpr17* leads to increase expression of *Olig2* and *Sox10*, However, the OPCs marker, *PDGFRα*, was not significantly different between the two genotypes. Which is consistent with previous analysis that loss of *Olig1* function has no obvious effect on the recruitment of progenitor cells but progenitors are impaired their ability to differentiate after demyelination^56^. *Olig2*, an oligodendrocyte lineage specific marker, expressed continuously throughout the lineage^57^. *Sox10* is an oligodendrocyte differentiation-promoting factor, required for terminal differentiation of the oligodendrocyte lineage^41–43^. The absence of *Gpr17* the enhanced oligodendrocyte differentiation was recruited toward demyelinated lesions, we hypothesized that this recruitment is correlate with *Sox10*.

It is critical to clearly understand the cellular and molecular mechanisms that regulate myelination in order to develop novel therapies to target remyelination^15,35,45,58,59^. We found the activated form of *Erk1/2* (p-*Erk1/2*) was increased in lesion areas at 14dpl, suggests that *Gpr17* negatively regulates the remyelination is correlate with Erk activation. In support of previous work showing that *Erk1/2* function as late-stage regulators of CNS myelination and that the control of myelin thickness is independent of oligodendrocyte development and initiation of myelin wrapping^46^, enhanced activation of *Erk1/2* in oligodendrocytes does not alter oligodendrocyte survival after LPC-induced demyelination^47^.

Activate *Erk1/2* in oligodendrocytes to directly or indirectly target a cohort of genes in myelinating oligodendrocytes that work together to upregulate the major myelin/cytoskeletal proteins above a basal level to promote the assembly and continued wrapping of the myelin sheath, thus increasing myelin thickness^46^. In the future, it will be important to define the upstream and downstream effectors of *Erk1/2* and to understand the interplay between *Gpr17* and *Erk1/2* in the integration of biosynthetic and cytoskeletal pathways that are pivotal for proper CNS myelination^46^. Endogenous and synthetic ligands of *Gpr17* have been developed to activate or antagonize *Gpr17* activity, respectively^19,22^. Being a membrane receptor, *Gpr17* represents an ideal ‘druggable’ target to be exploited for innovative regenerative approaches to acute and chronic CNS diseases. Therapies targeted to *Gpr17* through *Erk1/2* pathways may prove useful not only to drive accelerated remyelination, but also to generate thicker myelin sheaths potentially rendering CNS axons less vulnerable to future episodes of demyelination.

## Methods

### Animals

C57BL/6 mice were purchased and maintained in the Sichuan University Laboratory Animal Center. *Gpr17*^*−/−*^ mice were generated as described previously^23^ (Chen *et al*., 2009). All mice were maintained in the Sichuan University Laboratory Animal Center. The mice were housed in specific pathogen free (SPF) cages under standard laboratory conditions on a 12 h light/dark cycle with constant access to food and water. Animals of both sexes were used in the study (for spinal cord injury experiments, males only), and littermates were used as controls. There were no blinding assessors of experimental group at any stage of the experiments. All animal use and studies were approved by ethical committees of Sichuan University and by the Institutional Animal Care and Use Committee of Sichuan University. All experiments were performed in accordance with relevant guidelines and regulations.

### LPC-induced demyelinating injury in the spinal cord

LPC-induced demyelination was carried out in the ventrolateral spinal white matter of 8-week-old male mice. Anesthesia was induced and maintained by peritoneal injection of a mixture of ketamine (90 mg per kilogram body weight) and xylazine (10 mg per kilogram body weight). We make a vertical incision (about 1.5 cm) over the laminectomy site spanning from about thoracic vertebrae T8 to T13.Lift the skin, we can see the ribs. By tracing the ribs backward, we identify T10-T12. After exposing the spinal vertebrae at the level of T10–T12, meningeal tissue in the intervertebral space was cleared and the dura mater was pierced with a dental needle. One percent LPC (L-a-lysophosphatidylcholine, Sigma; 0.5 μl) via a Hamilton syringe attached to a glass micropipette was injected into the ventrolateral white matter via a stereotactic apparatus. Inject LPC at a rate of 1 μl/5 seconds. After injecting, wait 10 seconds and retract the needle. Importantly, charcoal was used to mark the site of LPC injection so that the area of tissue at the lesion center could be unambiguously identified even after remyelination was complete.Spinal cord tissues carrying the lesions were collected at time points as follows: 7 dpl, representing peak OPC recruitment^60,61^, 14 dpl, representing OL differentiation and new myelin sheath formation^62^, and 28 dpl, representing new myelin sheath formation (at least 9 mice per control and mutant groups were used for each time point analysis).

### Tissue processing and histochemistry

Animals were anesthetized and perfused transcardially with PBS briefly, followed by 4% (w/v) paraformaldehyde (PFA,Sigma-Alorich,441244) in sodium phosphate buffer (pH 7.4), for perfusion and immersion fixation is Formaldehyde which is dissolved PFA. Tissues were postfixed for 2 hours before cryoprotection with 30% sucrose in PBS overnight. Unfixed tissues were used for Quantitative real-time PCR and Western Blot. The tissue surrounding the injection site was dissected. The tissue processing and immunohistochemical staining procedures were performed as described previously^63^. Briefly, for tissue immunostaining, 18 μm cryosections were incubated overnight in primary antibodies diluted in block solution (PBS with 5% v/v normal goat serum (Sigma-Aldrich, St Louis) and 0.3% v/v Triton X-100). After washing with PBS, sections were then incubated overnight at 4 °C with corresponding Cy2 or Cy3 fluorophore-conjugated secondary antibodies (Jackson ImmunoResearch). Secondary antibodies were used at 1:1000. For BrdU staining, tissue sections were denatured with 0.1 N HCl for 1 hour in a 37 °C water bath. After denaturation, sections were neutralized with 0.1 M Borax pH 8.5 (Sigma-Aldrich, St Louis) for 10 min. Sections were washed with 0.3% Triton X-100/1-PBS (wash buffer) for three times and blocked with 5% normal donkey serum (Sigma-Aldrich) containing wash buffer for 1 hour at room temperature. Mouse anti-BrdU (BD Bioscience, 550891, 1:500) antibody was used to label BrdU overnight at 4 °C. Samples were mounted in Fluoromount G (SouthernBiotech) for fluorescent microscopy. For BrdU incorporation analysis, control and *Gpr17−/−* littermates were injected with BrdU (Sigma-Aldrich) (100 mg per kilogram body weight) 2 hours before anaesthesia. Primary antibodies used were as follows: Olig2 (Millipore,AB9610, 1:1,000), BrdU (BD Bioscience, 550891, 1:500), Sox10(Santa Cruz, sc-17343, 1:300),MBP(Abcam,ab40390,1:400). We define the lesion border by the increased cell density, shown by DAPI staining. Cell counted using ImageJ (National Institutes of Health).

### RNA *in situ* hybridization

RNA *in situ* hybridization was performed using digoxigenin-labelled riboprobes as described previously^64,65^. Briefly, following pretreatments (Proteinase K, postfixation, acetic anhydride), 18 μm cryosections were then prehybridized for 3–4 hours at 65 °C. Hybridization was carried out for 16 hours using 1–2 mg/ml of probe, in plastic slide mailers containing sufficient probe solution to immerse the part of the slides containing the sections. Probes used in this way could be re-used up to six or seven times without appreciable loss of signal. The alkaline phosphatase reaction product was developed using the NBT/BCIP reagents. Sections were prewashed with levamisole, and development was carried out for 2–20 hours depending upon the abundance of the target mRNA. Wash buffers containing CHAPS detergent were re-used to economize. Probes used for *in situ* hybridization were: *Mbp* and *Plp*, the *Plp* probe also recognizes *DM-20*.

### Western Blot Analysis

The tissues or cells were homogenized in lysis buffer (50 mM Tris, pH 7.4, 150 mMNaCl, 1 mM EDTA, 1% Triton X-100, 0.1% SDS) containing protease inhibitors (Roche Applied Science). The lysates were clarified by centrifugation at 13,000 × g at 4 °C for 20 min, and the supernatants were collected and normalized for protein concentration. Proteins were separated by 8% SDS-PAGE and transferred onto polyvinylidene difluoride membranes (Immobilon-P, Millipore). After blocking with PBS containing 5% skim milk and 0.05% Tween 20, the membranes were incubated with primary antibodies.For detection, a fluorescence-conjugated secondary antibody and an electrogenerated chemiluminescence system (GE Healthcare) were used. The membrane was exposed to an imaging system (LAS-3000, Fujifilm) according to the manufacturer’s specifications. The protein bands were quantified using ImageJ 1.44p software. The following antibodies were used: rabbit anti-p-ERK1/2 (1:1000,Santa Cruz, sc-7383); rabbit anti-ERK1/2 (1:1000,Santa Cruz,sc-292838), rabbit anti-GAPDH (1:5000, Abcam, ab128915). Horseradish peroxidase-conjugated rabbit IgG-specific (1:5000, Cell Signaling Technology, 7074 S) were used for secondary antibodies. Full-length gel is available at Supplementary data.

### Electron microscopy

The spinal cord regions from 8-week-old *Gpr17*^*−/−*^ or control mice were dissected and fixed in a solution of 2% paraformaldehyde, 2% glutaraldehyde (v/v), and 0.1 m cacodylic acid, pH 7.2, and processed for electron microscopy as described previously^23^ (Chen *et al*., 2009). Sections of 1 μm were cut, stained with toluidine blue, and examined by light microscopy, from which remyelination was identified using standard morphological criteria. G-ratio calculations of axons in the area of interest were calculated by dividing the diameter of an axon by the diameter of the axon plus the associated myelin sheath. A total of 120 axons of 1 μm diameter for each group of 3–4 animals were used. Images of transverse ultra-thin sections through the lesion were observed at a magnification of x10000 and analyzed using Image J. For remyelination analysis, the number of remyelinated axons was counted, excluding non-myelinated and non-demyelinated axons (G-ratio ≤ 0.55). Digitized and calibrated images were analyzed using ImageJ (National Institutes of Health). Overall G ratio was compared by unpaired t-test. Statistical significance was set at P < 0.05.

### Quantitative real-time PCR analysis

RNAs were isolated with Trizol (Invitrogen Inc.) from snap-frozen tissues. Reverse transcription was performed with the cDNA Reverse Transcription Kit (Bio-Rad) with iQ SYBR Green Supermix (170–8880). qRT–PCR was carried out using the Bio-Rad CFX96 Real-Time System using GAPDH as an internal control. Each analysis was performed in triplicates and the results were normalized to GAPDH for each sample. The qRT–PCR primer sequences are listed in Supplementary Table 1. Relative expression was calculated using Comparative Ct method^66^.

### Statistical analysis

The data for two-group comparisons were analyzed for statistical significance using two-tailed Student’s t tests. Error bars represent SEM. Values of p < 0.05 were considered significant.

### Data availability statement format guidelines

The datasets generated during and/or analyzed during the current study are available from the corresponding author on reasonable request.

## Electronic supplementary material

Supplementary Figures
