# Supplementary material for: G-Protein-Coupled Receptor Gpr17 Regulates Oligodendrocyte Differentiation in Response to Lysolecithin-Induced Demyelination
Source: Sci Rep. 2018 Mar 14;8:4502. doi: 10.1038/s41598-018-22452-0 (PMC5852120; doi:10.1038/s41598-018-22452-0)
Supplement: Supplementary file 1 — Supplementary Figures [file 41598_2018_22452_MOESM1_ESM.pdf]

G-Protein-Coupled Receptor Gpr17 Regulates Oligodendrocyte Differentiation in Response to Lysolecithin-Induced Demyelination

Changqing Lu[1,2, #], Lihua Dong[2, #],Hui Zhou[3, #],Qianmei Li[3],Guojiao Huang[3],Shu jun Bai,[3]and Linchuan Liao[1]*

[1]Department of Forensic Analytical Toxicology, West China School of Preclinical and Forensic Medicine, Sichuan University, Chengdu, 610041, Sichuan, China.

[2]Department of Anatomy, Preclinical and Forensic Medical Institute, Sichuan University, Chengdu, 610041, Sichuan, China.

[3]Department of Pediatrics, West China Second University Hospital, Sichuan University, Chengdu, Sichuan Province, China.

# These authors contributed equally to this work. Correspondence and requests for materials should be addressed to C.L (email: changing_lu@126.com)or L.L. (email: linchuanliao@scu.edu.cn)

Supplementary Figure S1 Immunostaining of *MBP* in the contralateral areas of the spinal cord of the injected animals.
Scale bar, (A)100μm;

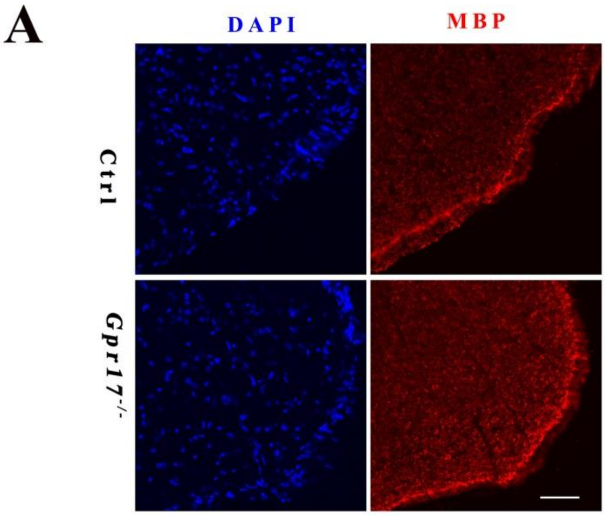

Supplementary Figure S2. Original full-length western blot image of Figures 5C using antibodies of phosphorylated Erk1/2 (p-Erk1/2) and GAPDH.
Western blot analysis of pERK1/2 expression in lesion regions at 14 dpl in spinal cords of 8-week-old control and *Gpr17*$^{-/-}$ mice; n=4 animals for each genotype. Lane 1,2,6,8 were Gpr17$^{-/-}$

mice; Lane 3,4,7,9 were control mice; M was protein molecular weight marker.

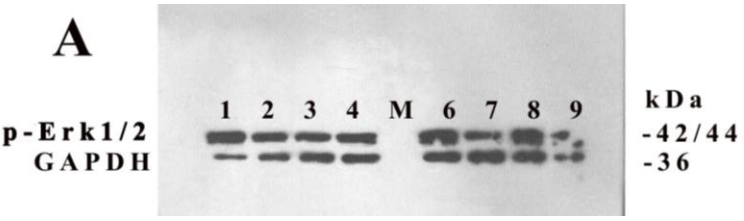

Supplementary Figure S3. Original full-length western blot image of Figures 5 using antibodies of Erk1/2 and GAPDH.

A: Western blot analysis of Erk1/2 expression in lesion regions at 14 dpl in spinal cords of 8-week-old control and *Gpr17*$^{-/-}$ mice; n=4 animals for each genotype. Lane 1,2,6,8 were Gpr17$^{-/-}$ mice; Lane 3,4,7,9 were control mice; M was protein molecular weight marker.

B:The relative expression of ERK1/2 between control and Gpr17$^{-/-}$mice. Student's t-test. P>0.05.

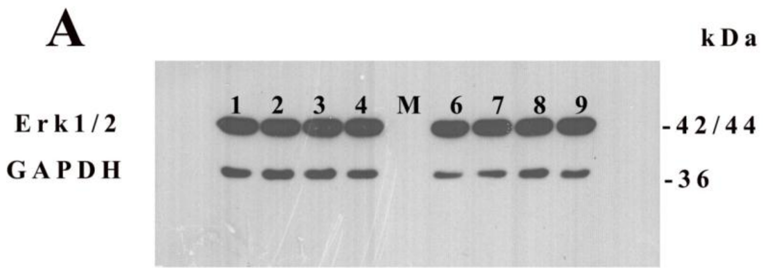

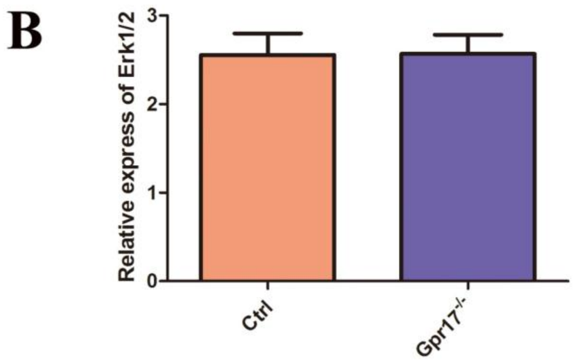

Supplementary Table T1. Primer Sets for in situ hybridization and Quantitative RT-PCR

Gene	Forward	Reverse
Mbp	5'- GAGGCCTGGATGTGATGG-3'	5'-GGGGAACAAGTCAGGGCT-3'
Plp	5'-GGGGATGCCTGAGAAGGT-3'	5'-TGTGATGCTTTCTGCCCA-3'
Pdgfrα	5'-TGGCAAAGAACAACCTCAG-3'	5'-CGATAACCCTCCAGCGAAT-3'
b-actin	5'-GAGGTATCCTGACCCTGAAGTA-3'	5'-CACACGCAGCTCATTGTAGA-3'
